# CMTM1_v17 is associated with chemotherapy resistance and poor prognosis in non-small cell lung cancer

**DOI:** 10.1186/s12957-016-1094-z

**Published:** 2017-01-28

**Authors:** Jiahui Si, Panpan Zhang, Dan Tian, Xing Wang, Yuanyuan Ma, Jianzhi Zhang, Lu Wang, Yue Yang

**Affiliations:** 1 Department of Thoracic Surgery II, Key laboratory of Carcinogenesis and Translational Research (Ministry of Education/Beijing), Peking University Cancer Hospital and Institute, Beijing, 100142 China; 2 Department of Immunology, School of Basic Medical Science, Health Science Center, Peking University, Beijing, 100142 China

**Keywords:** Non-small cell lung cancer, Neoadjuvant chemotherapy, CMTM1_v17, Chemoresistance, Prognosis

## Abstract

**Background:**

Considering neoadjuvant chemotherapy (NAC) prior to surgery could shrink and reduce the primary tumor and distant micro-metastases to reduce the high relapses rates, NAC has been an accepted therapeutic management for patients with non-small cell lung cancer (NSCLC). CMTM1_v17 is highly expressed in human testis tissues and solid tumor tissues but relatively low expression was obtained in the corresponding normal tissues. This study aims to investigate the significance of CMTM1_v17 in NSCLC and its association with platinum-based NAC efficacy.

**Methods:**

31 pairs of tumor tissues before and after NAC and 78 resected tumor tissues after NAC were utilized for immunohistochemistry (IHC) staining of CMTM1_v17 protein. The correlation between CMTM1_v17 expression and chemotherapy efficacy was analyzed. The prognostic value of CMTM1_v17 index for disease-free survival (DFS) and overall survival (OS) was analyzed using Kaplan-Meier survival and multivariable Cox regression.

**Results:**

CMTM1_v17 expression was related to treatment effect and outcome in tumor tissues after NAC not before NAC from 31 cases of NSCLC. We identified that high CMTM1_v17 expression was associated with low objective remission rate (ORR) (*P* = 0.008) and poor prognosis (the median OS: 35.1 months vs 65.6 months, *P* = 0.0045; the median DFS: 17.27 months vs 35.54 months, *P* = 0.0207) in the 31 patients. Next, we detected CMTM1_v17 expression to confirm correlation between this protein status and clinical characteristics in 78 NSCLC patients with NAC treatment. The upregulation of CMTM1_v17 had a higher SD rate (*P* = 0.007) and worse outcome (the median OS: 41.0 months vs 80.6 months, *P* = 0.0028; the median DFS: 33.4 vs 64.8 months, *P* = 0.0032). COX multivariate analysis indicated that CMTM1_v17 is an independent prognostic risk factor on patients who have received NAC (OS: HR = 3.642, *P* = 0.002; DFS:HR = 3.094, *P* = 0.002).

**Conclusions:**

CMTM1_v17 expression is significantly associated with chemoresistance and poor prognosis of the early stage NSCLC patients who have received NAC.

## Background

Lung cancer remains the leading cause of cancer-related mortality for both men and women in China [1]. Non-small cell lung cancer (NSCLC) is the most commonly diagnosed lung cancers, accounting for up to 80% of all histological subtype of lung cancer [2]. Patients with NSCLC in stage IIB/IIIA generally have unfavorable prognosis [3]. The neoadjuvant chemotherapy (NAC) prior to surgery has been reported to shrink and reduce the primary tumor and distant micro-metastases, thereby reducing the high relapses rates [4]. In our previous study, platinum-based NAC was demonstrated to significantly increase disease-free survival (DFS) time for patients with stage IIB-IIIA central disease [5]. Factors such as good pathological response and shrinkage of mediastinal nodal have been reported to be related to NAC efficacy in several randomized trials [6–8], but biological molecular markers’ variation for the patients with NAC have not been fully investigated.

The CMTM1 gene is a member of the chemokine-like factor superfamily (CKLFSF). CMTM1_v17 is one of the 23 variants of CMTM1. The protein is composed by 149 amino acids [9]. In humans, CMTM1_v17 apparently exhibits a tissue-specific expression. High level of CMTM1_v17 is expressed in testicles and prostate tissues but low or undetectable level is found in many normal tissues [10]. Previous investigation demonstrated that CMTM1_v17 expressed in various types of solid tumors (breast cancer, kidney cancer, lung cancer, liver cancer, and ovarian cancer) and could promote the proliferation and lead to partial resistance to tumor necrosis factor-α (TNF-α) induced apoptosis via activation of NF-κB signaling pathway in breast cancer [11]. However, little is known about the significance of CMTM1_v17 in NSCLC and its association with platinum-based NAC efficacy.

In the present study, we investigated the expression of CMTM1_v17 in tumor tissues of NSCLC patients before and after NAC using IHC to determine whether the expression of CMTM1_v17 could have the predictive value for the rational application of NAC and prognostic value in NSCLC patients.

## Methods

### Patients

A total of 78 NSCLC patients, who had been treated with NAC and surgery from July 2006 to April 2012, were enrolled in our study. The median age at diagnosis was 56 years (range, 38 to 75 years). Patients were selected for our study based on the following features: (1) all patients had been diagnosed with NSCLC by pathological diagnosis; (2) presence of central disease with T2bN1, T3 or T4 N0, or locally advanced disease with T1 to T3 N2; (3) patients had received at least 2 cycles of platinum-based chemotherapy followed by surgery; (4) had no advanced disease such as N3 or M1; (5) had not received radiotherapy; (6) clinical variables were recorded in detail including gender, age, histology, smoking, disease location, chemotherapy regimen, clinical response, TNM stage, disease recurrence, and survival. Matched biopsy and surgical resection samples were collected in 31 of 78 NSCLC patients before and after neoadjuvant treatment.

**Table 1:** Patient characteristics enrolled in this study (*n* = 31)

Variable	Number	Percentage(%)
Total	31	100
Age (years)		
≤ 55	16	51.7
> 55	15	48.4
Gender		
Male	25	80.6
Female	6	19.4
Smoking		
Non-smoker	8	25.8
Smoker	23	74.2
Histology		
Adenocarcinoma	11	35.5
Non-adenocarcinoma	20	64.5
Histologic grading		
Poorly	16	51.6
Moderate and well	15	48.4
Venous invasion		
Negative	23	74.2
Positive	8	25.8
Pathological stage		
I/II	17	54.8
III	14	45.2

Patients’ clinical and pathological features were derived from the clinical database established in 2000. Pathologic staging of lung cancer was reviewed and classified according to the 2009 International Union Against Cancer–American Joint Committee on Cancer–TNM system (version 7) [12]. Histologic subtypes were based on the World Health Organization (WHO) criteria [13].

The study was conducted with the approval of the Institutional Ethic Committee at Peking University Cancer Hospital. Lung tumor samples were analyzed with the agreement of the patients who have signed informed consent.

### IHC and quantification of CMTM1_v17 positive tumor cells

Formalin-fixed and paraffin-embedded primary lung cancer samples were acquired from the Department of Pathology, Peking University, under approval from the Ethical Committee. For CMTM1_v17 staining, sections (4 um) were routinely processed and stained using mouse anti-CMTM1_v17 (1:800 dilution; acquired from Wang Lu; School of Basic Medical Sciences, Health Science Center, Peking University, Department of Immunology) polyclonal antibody followed by incubation with HRP-conjugated goat anti-mouse secondary antibody (Sigma-Aldrich, Poole, Dorset, UK). For determination of immunoreactivity for CMTM1_v17, cytosolic staining of yellowish or brownish granules was graded as follows: (a) for background staining; (b) for negative staining; (c) for moderate staining, and (d) for strong staining. In addition, positive staining areas in the entire tissue section were graded as follows: 0 for <5%; 1 for 5–25%; 2 for 26–50%, and 3 >50%. When combining these two parameters, 0–1 and >1 were considered CMTM1_v17 low expression and CMTM1_v17 high expression, respectively.

### Statistical analysis

All statistical analyses were performed with the Statistical Package for Social Sciences software version 17.0 (SPSS17.0). The correlation between CMTM1-v17 expression and clinicopathologic variables was assessed using Pearson’s chi-squared test or Fisher’s exact test. Disease-free survival (DFS) was defined as the time from surgery to tumor recurrence, death or the date of the last follow-up. The survival rates were estimated using the Kaplan-Meier method, and the differences in survival between the subgroups were compared using the log-rank test. A multivariate analysis was conducted to study the prognostic value of CMTM1_V17 using the Cox proportional-hazard model. A two sided *P* value of less than 0.05 was considered to be statistically significant at all situations.

## Results

### CMTM1_v17 variation in the NSCLC cancers before and after NAC

**Fig. 1:**
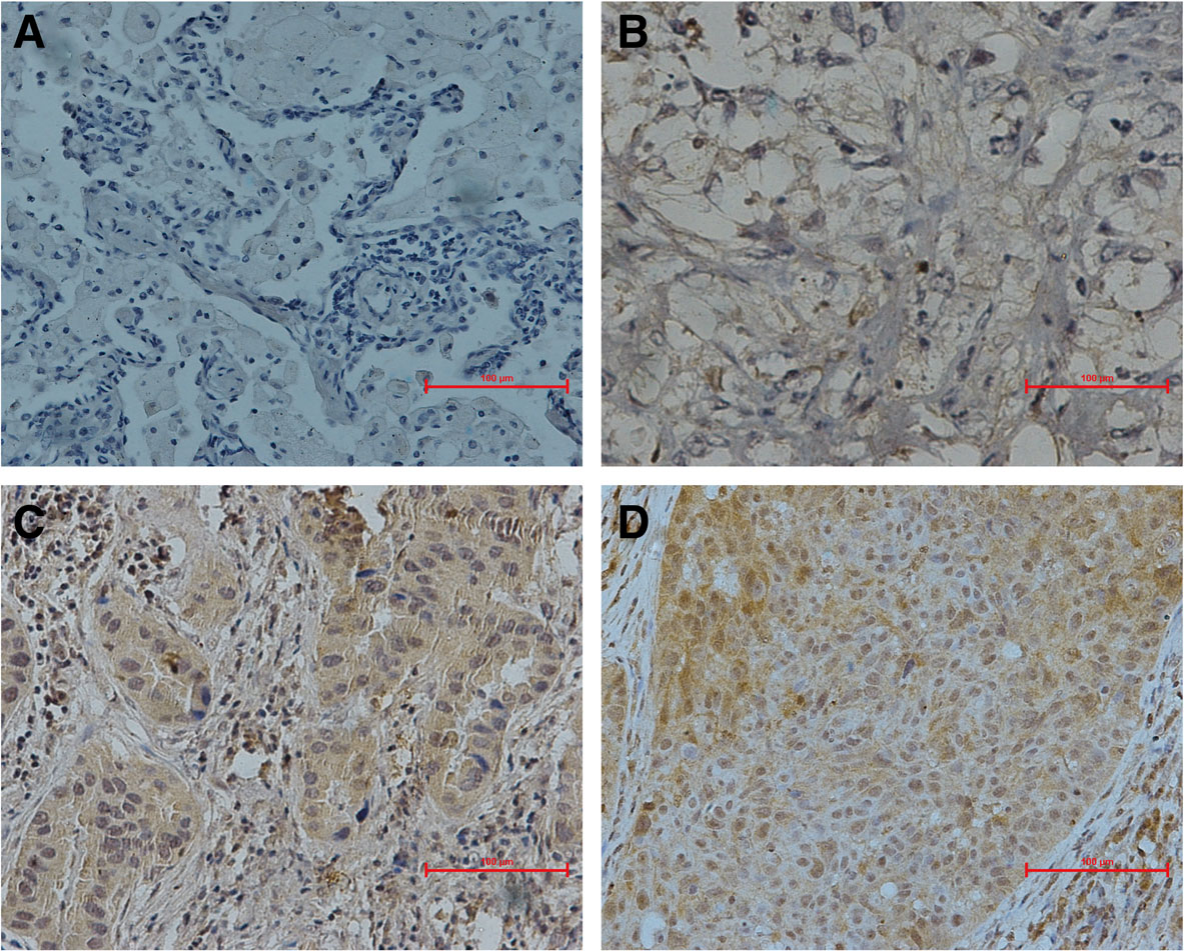
Immunohistochemical staining for CMTM1_v17. **a**, **b** The low expression of CMTM1_v17 in non-small cell lung cancer (NSCLC) primary tumor; (**c**, **d**) The high expression of CMTM1_v17 in NSCLC primary tumor; the magnification was ×200

Thirty-one NSCLC patients were recruited into the study from July 2006 to April 2012. Patients’ sociodemographic, pathologic, and clinical characteristics were listed in Table 1. Tumor core biopsies were successfully obtained in 31 cases of these patients before NAC. Figure 1 illustrated representative CMTM1_v17 IHC staining. Lung cancer tissues showed strong and diffuse cytoplasmic staining of CMTM1_v17. In 31 NSCLC patients, the correlation of CMTM1_v17 expression in tumor tissues pre- and post-NAC according to various prognostic groups was shown in Table 2. There was no significant correlation of CMTM1_v17 expression with any other parameters, such as patients’ age, gender, smoking history, histology, pathological stage, though there were more cases with CMTM1_v17 high expression after NAC in pathological stage III (71.4%) compared to pathological stage I/II (41.2%).

**Table 2:** Patients’ characteristics and levels of CMTM1_v17 expression pre- and post-NAC (*n* = 31)

Variable	Pre-NAC			Post-NAC		
	CMTM1_v17 expression no. (%)		*P* value	CMTM1_v17 expression no. (%)		*P* value
	High	Low		High	Low	
Age			0.200			0.552
≤ 55	7(43.8)	9(56.3)		7(43.8)	9(56.2)	
> 55	10(66.7)	5(33.3)		5(33.3)	10(66.7)	
Gender			0.763			0.791
Male	10(40.0)	15(60.0)		14(56.0)	11(44.0)	
Female	2(33.3)	4(66.7)		3(50.0)	3(50.0)	
Smoking history			0.935			0.75
Non-smoker	3(37.5)	5(62.5)		4(50.0)	4(50.0)	
Smoker	9(39.1)	14(60.9)		13(56.5)	10(43.5)	
Histology			0.332			0.981
Adenocarcinoma	3(27.3)	8(72.7)		6(54.5)	5(45.5)	
Non-adenocarcinoma	9(45.0)	11(55.0)		11(55.0)	9(45.0)	
Histologic grading			0.379			0.576
Poorly	5(31.3)	11(68.7)		8(50.0)	8(50.0)	
Moderate and well	7(46.7)	8(53.3)		9(60.0)	6(40.0)	
Venous invasion			0.355			0.75
Negative	10(43.5)	13(56.5)		13(56.5)	10(43.5)	
Positive	2(25.0)	6(75.0)		4(50.0)	4(50.0)	
Pathological stage			0.756			0.092
I/II	7(41.2)	10(58.8)		7(41.2)	10(58.8)	
III	5(35.7)	9(64.3)		10(71.4)	4(28.6)	

*P* value was calculated using Pearson’s χ2 test

*NAC* neoadjuvant chemotherapy

**Fig. 2:**
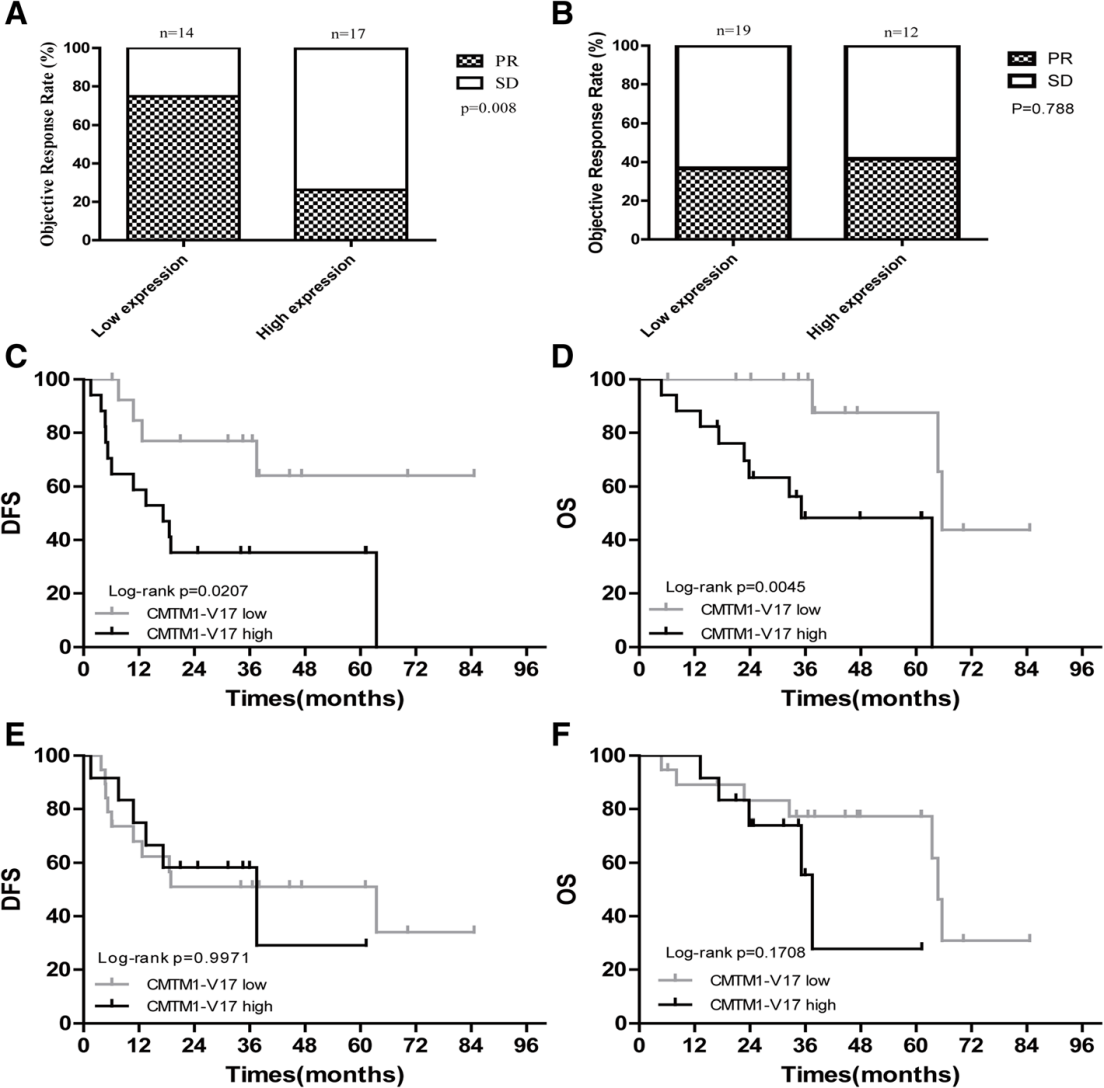
Chemotherapy efficacy and prognosis analysis according to CMTM1_v17 expression pre- and post-chemotherapy in 31 patients. **a** At the end of NAC, patients with low expression of CMTM1_v17 were sensitive to chemotherapy, with high PR rates compared to those who were CMTM1_v17 high expression (*P* = 0.008). **b** Before NAC, there was no significant difference in chemotherapy efficacy in CMTM1_v17 high vs CMTM1_v17 low groups (*P* = 0.788). These Kaplan-Meier curves illustrate the prognostic significance of CMTM1_v17 pre- and post-NAC in 31 NSCLC patients. **c**, **d** At the end of NAC, patients with low expression of CMTM1_v17 had much better prognosis compared to those who were CMTM1_v17 high expression. **e**, **f** Before NAC, there was no significant difference in DFS and OS in CMTM1_v17 high vs CMTM1_v17 low groups. *OS*, overall survival; *DFS*, disease-free survival; *NAC*: neoadjuvant chemotherapy

To assess the clinical significance of CMTM1_v17 expression in 31 NSCLC, we analyzed the relationship between CMTM1_v17 expression and NAC efficacy. The results showed that the expression of CMTM1_v17 in tumor tissues after NAC strongly correlated with NAC efficacy, with partial response (PR) rates of only 25.0% in CMTM1_v17 high expression tumors compared to 73.7% in CMTM1_v17 low expression tumors (*P* = 0.008, Fig. 2a). However, there was no significant association between CMTM1_v17 expression in tumor tissues before NAC and chemotherapy response (*P* = 0.788, Fig. 2b).

Moreover, we identified that high expression level of CMTM1_v17 in tumor tissues after NAC strongly was correlated with poor survival (DFS: *P* = 0.0207, Fig. 2c; OS: *P* = 0.0045, Fig. 2d). But the expression of CMTM1_v17 in tumor tissues before NAC was not associated with DFS (*P* = 0.9971, Fig. 2e) and OS (*P* = 0.1708, Fig. 2f). That is to say, high expression level of CMTM1_v17 in tumor tissues after NAC was associated with chemoresistance and poor prognosis, while CMTM1_v17 expression in tumor tissues before NAC was not correlated with chemotherapy response and survival.

Then univariate and multivariate analyses were performed to identify clinicopathological factors influencing the OS and 5-year DFS according to the Cox proportional hazard model, and the log-rank test was used to compare the two groups. Among the clinical factors, high expression level of CMTM1_v17 post-NAC was significantly correlated with a shorter OS (*P* = 0.021, HR = 0.074). Older age (>55 years) and pathological stage III were significantly correlated with shorter DFS (Tables 3 and 4).

**Table 3:** Univariate analysis of clinicopathological factors for OS and DFS in patients with NSCLC (*n* = 31)

Variables	OS			DFS		
	HR	95%CI	*P* value	HR	95%CI	*P* value
Age						
≤ 55 vs > 55	0.3624	0.1117–1.175	0.0908	0.3255	0.1182–0.8964	**0.0299**
Gender						
Male vs female	0.7042	0.1642–3.020	0.6368	0.4944	0.1269–1.927	0.3101
Smoking						
Non-smoker vs smoker	1.698	0.4399–6.552	0.4423	1.672	0.5113–5.469	0.395
Histology						
AD vs non-AD	0.9252	0.2659–3.219	0.9027	0.4909	0.1660–1.452	0.1985
Histologic grading						
Poorly Moderate and well	2.005	0.6213–6.473	0.2445	2.079	0.7724–5.595	0.1474
Venous Invasion						
Negative vs positive	1.352	0.3956–4.619	0.6306	1.237	0.4191–3.653	0.6999
Pathological stage						
I/II vs III	0.3045	0.09458–0.9802	**0.0462**	0.3044	0.1083–0.8553	**0.024**
Pre-NAC CMTM1_v17						
Low vs high	0.3753	0.09230-1.526	0.1708	1.002	0.3533–2.842	0.9971
Post-NAC CMTM1_v17						
High vs low	0.1587	0.04462–0.5647	**0.0045**	0.3099	0.1149–0.8360	**0.0207**

*P* value was calculated using a two-sided log-rank test.

*AD* adenocarcinoma, *NAC* neoadjuvant chemotherapy, *HR* hazard ratio (log-rank), *CI* confidence interval.

Bold values are significant (*p* < 0.05)

**Table 4:** Multivariable analysis of OS and DFS in patients received NAC (*n* = 31)

Variable	OS			DFS		
	HR	95%CI	*p* value	HR	95%CI	*p* value
Post-NAC CMTM1_v17						
Low vs high	0.074	0.008–0.670	**0.021**	0.455	0.136–1.517	0.12
Pathological stage						
I/II vs III	0.295	0.082–1.049	0.059	0.319	0.107–0.951	**0.04**
Age						
≤ 55 vs > 55				0.291	0.923–0.915	**0.035**

*P* value was calculated using a two-sided log-rank test.

*OS* overall survival, *DFS* disease-free survival, *HR* hazard ratio (log-rank), *CI* confidence interval.

Bold values are significant (*P* < 0.05)

### CMTM1_v17 expression level was related to chemoresistance and prognosis after NAC

Due to the small sample size of our study, COX multivariate analysis suggested that CMTM1_v17 was not associated with DFS in 31 NSCLC patients. Therefore, we added 47 NSCLC patients with NAC prior to surgery during that same period to our study. There was no significant difference between age, gender, smoking history, pathological stage, differentiation, among those patients (Table 5). Then, the relationship between CMTM1_v17 expression and chemoresistance and survival were analyzed in 78 patients who have received NAC.

**Table 5:** Patient’s characteristic, overall and according to the time of enrollment

Variable	Overall	Original patients group	Added patients group	*P* value
	no. (%)	no. (%)	no. (%)	
Total	78(100%)	31(39.7%)	47(60.3%)	
Age				0.246
≤ 55	32(41.1%)	16(50.0%)	16(50.0%)	
> 55	46(58.9%)	15(32.6%)	31(67.4%)	
Gender				0.793
Male	64(82.1%)	25(39.1%)	39(60.9%)	
Female	14(17.8%)	6(42.9%)	8(57.1%)	
Smoking history				0.978
Non-smoker	20(25.6%)	8(40.0%)	12(60.0%)	
Smoker	58(74.4%)	23(39.7%)	35(60.3%)	
Histology				0.661
Adenocarcinoma	30(38.5%)	11(36.7%)	19(63.3%)	
Non-adenocarcinoma	48(61.5%)	20(41.7%)	28(58.3%)	
Histologic grading				0.549
Poorly	37(47.4%)	16(43.2%)	21(56.8%)	
Moderate and well	41(52.6%)	15(36.6%)	26(63.4%)	
Venous invasion				0.347
Negative	62(79.5%)	23(37.1%)	39(62.9%)	
Positive	16(20.5%)	8(50.0%)	8(50.0%)	
Pathological stage				0.744
I/II	41(52.6%)	17(41.5%)	24(58.5%)	
III	37(47.4%)	14(37.8%)	23(62.2%)	

*P* value was calculated using Pearson’s χ2 test

**Fig. 3:**
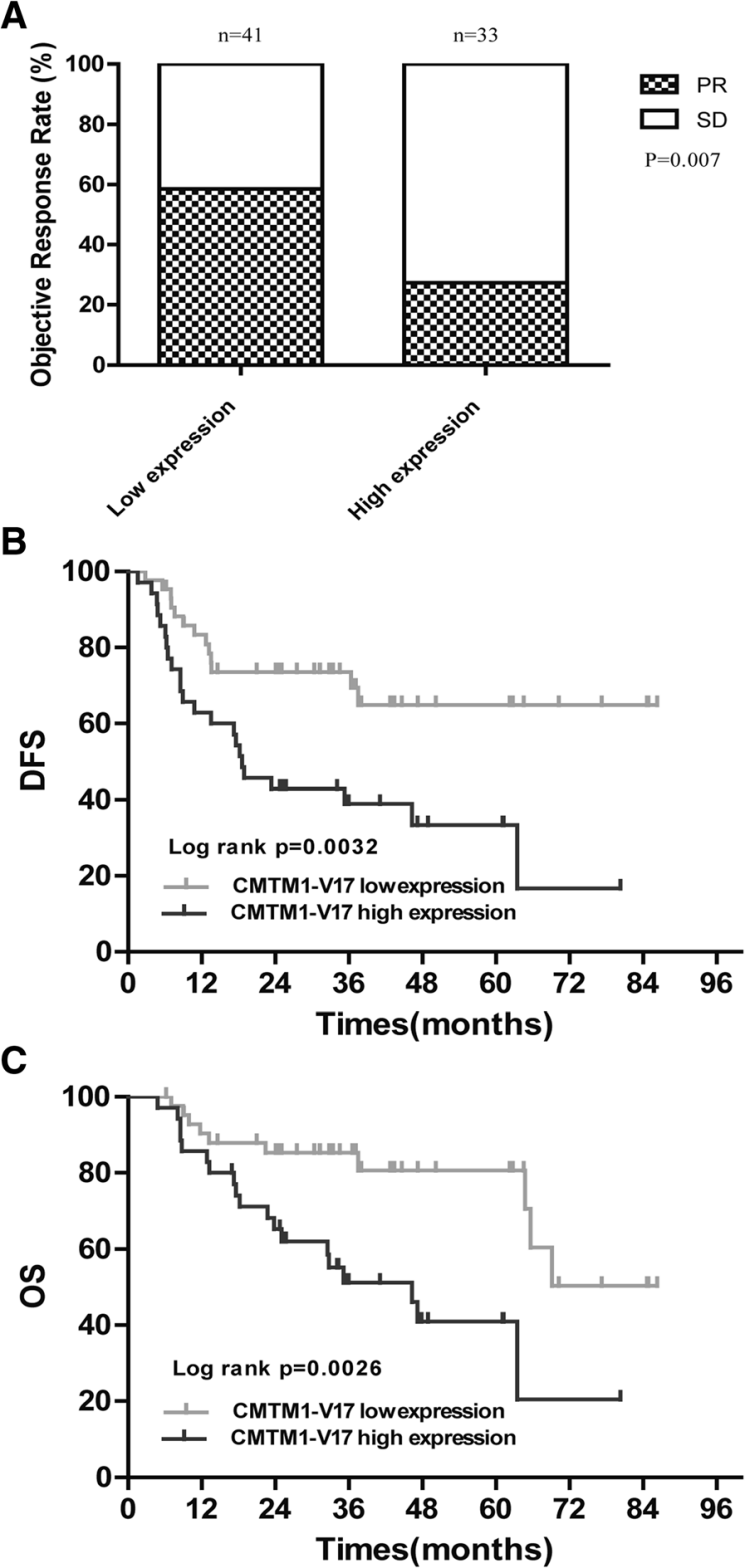
Chemotherapy efficacy and prognostic significance of CMTM1_v17 expression in patients with NAC treatment. **a** PR rates were higher in patients with CMTM1_v17 low expression than those with CMTM1_v17 high expression. **b**, **c** Patients with low expression of CMTM1_v17 had significantly better DFS and OS compared to those with high expression of CMTM1_v17 after NAC. *NAC* neoadjuvant chemotherapy; *PR* partial response; *OS* overall survival; *DFS* disease-free survival

Among 78 patients with NAC, 4 patients could not be evaluated the response to NAC because of the absence of clinical pathological features. The correlation of CMTM1_v17 expression and chemotherapy efficacy was analyzed on the remaining 74 patients with NAC treatment. The results also supported above findings, as summarized in Fig. 3. A high CMTM1_v17 expression level in the lung cancer cells was significantly correlated with chemoresistance (*P* = 0.007, Fig. 3a) and inferior DFS (*P* = 0.0032, Fig. 3b) and OS (*P* = 0.0026, Fig. 3c), compared with patients with a low CMTM1_v17 expression level. The association of CMTM1_v17 expression with patient clinicopathological parameters was shown in Table 6.

**Table 6:** Patients’ characteristics and levels of CMTM1_v17 expression in patients received NAC (*n* = 78)

Variable	CMTM1_v17 expression no. (%)		*P* value
	High	Low	
Age			0.315
≤ 55	13(40.6)	19(59.4)	
> 55	22(47.8)	24(52.2)	
Gender			0.308
Male	27(42.2)	37(57.8)	
Female	8(57.1)	6(42.9)	
Smoking history			0.593
Non-smoker	10(50.0)	10(50.0)	
Smoker	25(43.1)	33(56.9)	
Histology			0.352
Adenocarcinoma	13(43.3)	17(56.7)	
Non-adenocarcinoma	22(45.8)	26(54.2)	
Histologic grading			0.465
Poorly	15(40.5)	22(59.5)	
Moderate and well	20(48.8)	21(51.2)	
Venous invasion			0.506
Negative	29(46.8)	33(53.2)	
Positive	6(37.5)	10(62.5)	
Pathological stage			0.856
I/II	18(43.9)	23(56.1)	
III	17(45.9)	20(54.1)	

*P* value was calculated using Pearson’s χ2 test.

*NAC* neoadjuvant chemotherapy

COX univariate and multivariate analysis suggested that CMTM1_v17 is an independent prognostic risk factor in 78 NSCLC patients who have received NAC (OS: HR = 3.642, *P* = 0.002; DFS: HR = 3.094, *P* = 0.002, Tables 7 and 8). Collectively, these results strongly indicate that CMTM1_v17 expression is directly associated with chemotherapy efficacy and prognosis for patients who have received NAC.

**Table 7:** Univariate analysis of clinicopathological factors for OS and DFS in patients received NAC (*n* = 78)

Variables	OS		DFS	
	HR(95% CI)	*P* value	HR(95% CI)	*P* value
Age	0.7963(0.3753–1.689)	0.5528	0.6167(0.3178–1.197)	0.1531
Gender	0.5719(0.2129–1.536)	0.2678	0.3019(0.1163–0.7836)	**0.0138**
Smoking history	1.648(0.7097–3.827)	0.2452	2.134(0.976–4.663)	0.0575
Histology	0.4711(0.2217–1.001)	0.0503	0.351(0.1761–0.6996)	**0.0029**
Histologic grading	1.425(0.6849–2.964)	0.3435	1.714(0.8874–3.312)	0.1086
Venous invasion	0.6148(0.2552–1.482)	0.2784	0.7369(0.3251–1.670)	0.4645
Pathological stage	0.3958(0.1894–0.8274)	**0.0137**	0.3351(0.1717–0.6540)	**0.0014**
CMTM1_v17 expression	0.3095(0.1442–0.6643)	**0.0026**	0.3655(0.1871–0.7142)	**0.0032**

*P* value was calculated using a two-sided log-rank test

*NAC* neoadjuvant chemotherapy, *HR* hazard ratio (log-rank), *CI* confidence interval.

Bold values are significant (*P* < 0.05)

**Table 8:** Multivariable analysis of OS and DFS in patients received NAC (*n* = 78)

Variable	OS			DFS		
	HR	95%CI	*P* value	HR	95%CI	*P* value
CMTM1_v17						
High vs low	3.642	1.594–8.324	**0.002**	3.094	1.507–6.350	**0.002**
Pathological stage						
III vs I/II	2.862	1.316–6.224	**0.008**	2.704	1.315–5.560	**0.007**
Gender						
Male vs female				1.026	0.399–2.636	0.958
Histology						
Adenocarcinoma vs non-Adenocarcinoma				2.038	0.879–4.727	0.097

*P* value was calculated using a two-sided log-rank test

*OS* overall survival, *DFS* disease-free survival, *HR* hazard ratio (log-rank), *CI* confidence interval.

Bold values are significant (*P* < 0.05)

## Discussion

The aim of this study was to investigate the significance of CMTM1_v17 in NSCLC and its association with platinum-based NAC efficacy. The expression status of CMTM1_v17 in patients with NSCLC before and after treatment with NAC was evaluated. We demonstrated that a high expression of CMTM1_v17 confers chemoresistance and poor clinical outcome in patients who have received NAC. To our knowledge, this is the first study to demonstrate the correlation between CMTM1_v17 expression and chemotherapy efficacy and prognosis.

Cisplatin was approved by FDA for treating testicular tumors and bladder cancers for the first time in 1978, and gradually employed in the treatment of multiple solid tumors. However, cisplatin has poor curative effect for the NSCLC disease compared with response of this drug on the other type of solid cancers. Cisplatin exerts anticancer effects mainly via the generation of DNA lesions followed by the activation of the DNA damage response and the induction of mitochondrial apoptosis [14, 15]. Previous investigations have demonstrated that TP53, BCL-2 family, caspase family, and MAPK family often influence cisplatin sensitivity in tumor cells via cell apoptosis [16–24].

The strong impact of CMTM1_v17 in promoting tumor cell proliferation and lead to partial resistance to TNF-α-induced apoptosis likely via activation of NF-κB signaling pathway has been validated in breast cancer [11]. Previous study revealed that TNF-α could increase the sensitivity of cancer cells to cisplatin-induced cell death by NF-κB signaling pathway [25]. Furthermore, the activation of the NF-κB pathway has also been reported to be associated with cisplatin resistance [26].Therefore, we developed a hypothesis that the high expression of CMTM1_v17 could promote chemoresistance. When analyzing chemotherapy efficacy in the correlation of the expression level of CMTM1_v17, patients with low CMTM1_v17 expression in the tumor tissues after NAC suggested higher PR rates than those with high CMTM1_v17 expression, which were confirmed in 78 NSCLC patients who have received NAC. However, the expression of CMTM1_v17 in the tumor tissues before NAC was not a predictor of response to chemotherapy. The above hypothesis was verified by the results that CMTM1_v17 high expression was correlated to chemoresistance in NSCLC patients treated with NAC. Our results are significantly important from the opinion that it could distinguish patients who are possibility to benefit from adjuvant chemotherapy towards personalized treatment.

Then the impact of CMTM1_v17 on prognosis was analyzed. Patients with low CMTM1_v17 expression in tumor tissues after NAC lived significantly longer than those with high CMTM1_v17 expression, but there is no association between CMTM1_v17 expression in tumor tissues before NAC and survival. These results identified that the expression level of CMTM1_v17 might be a prognostic marker only in NSCLC patients who have received NAC. These findings are similar to those of Alamgeer et al. [27], who reported that chemotherapy could affect the expression of ALDH1 in breast cancer, and the expression of ALDH1 at baseline does not impact the long-term prognosis, but that after chemotherapy is associated with prognosis. However, a larger study may be needed to prove this finding, and the molecular mechanisms are needed for further in vitro or in vivo investigations.

Surprisingly, our study found the variable expression of CMTM1_v17 in tumor tissues before and after NAC for patients with NSCLC. The phenomenon can be explained that during chemotherapy, tumor cells will redistribute and cells resisting to chemotherapy will gradually expand at the expense of their relatively chemosensitive counterparts, resulting in difference of gene expression before and after treatment. In addition, the changes of inflammatory states and tumor microenvironment regulated by inflammatory cytokines may further influence tumor genome [28–30].

## Conclusion

CMTM1_v17 expression is significantly associated with chemotherapy resistance and poor prognosis of the early stage NSCLC patients who have received NAC.

## Abbreviations

CKLFSF: Chemokine-like factor superfamily
CMTM1_v17: CKLF-like MARVEL transmembrane domain family 1-variant 17
DFS: Disease-free survival
HR: Hazard ratio
IHC: Immunohistochemistry
NAC: Neoadjuvant chemotherapy
NSCLC: Non-small cell lung cancer
ORR: Objective remission rate
OS: Overall survival
PR: Partial response
SD: Stable disease
